# 

**Published:** 1897-05

**Authors:** 


### CONTENTS.

ADDRESSES—

PRESIDENT'S ADDRESS, FORTY-EIGHTH ANNUAL SESSION OF THE MEDICAL ASSOCIATION OF GEORGIA, MACON, APRIL 21, 1897. By Geo. H. Noble, M.D., Atlanta, Ga.. 145

NINETEENTH CENTURY MEDICINE. By William Whatley Battey, M.D., Augusta, Ga. 152

ORIGINAL COMMUNICATIONS—

SOME SURGICAL OPERATIONS RECENTLY PERFORMED IN THE MACON HOSPITAL. By Howard J. Williams, A.M., M.D., Macon, Ga.... 160

THE RELATION OF THE STATE BOARD OF MEDICAL EXAMINERS TO THE MEDICAL PROFESSION OF THE STATE. By E. R. Anthony, M.D.. Griffin, Ga.... 169

LACTOPHENIN. By J. A. Childs, M.D., Atlanta, Ga.... 175

SOCIETY REPORTS—

MEDICAL ASSOCIATION OF GEORGIA.... 178

CORRESPONDENCE—

NEW YORK LETTER.... 189

CARD FROM DR. J. F. ALEXANDER.... 194

EDITORIAL—

THE MEDICAL ASSOCIATION'S MEETING AT MACON.... 195

SELECTIONS AND ABSTRACTS—

RHINOLOGY AND LARYNGOLOGY.... 198

MISCELLANEOUS.... 201

MEDICAL ITEMS.... 210

BOOK REVIEWS.... 214

MEDICAL ITEMS—CONTINUED.... x, xii, xiv, xvi, xvii, xviii, xix

ADVERTISERS' INDEX TO ADVERTISEMENTS.... xx

